# Untangling the Strands of Hairy Cell Leukemia: The Clinicopathological Spectrum over Eleven Years at a Tertiary Care Center

**DOI:** 10.15190/d.2023.5

**Published:** 2023-06-30

**Authors:** Varnika Rai, Poornima Manimaran, Anurag Saha, Vikas Kailashiya, Jyoti Sawhney, Sandeep Ramawat, Sneha Kakoty

**Affiliations:** ^1^Onco-Pathology Department, Gujarat Cancer & Research Institute, Ahmedabad, India; ^2^Department of Pathology, Institute of Medical Sciences, Banaras Hindu University, India; ^3^Medical Oncology Department, Gujarat Cancer and Research Institute, Ahmedabad, India

**Keywords:** Hairy cell leukemia, BRAF V600E mutation, Cladribine, Flowcytometry, Bone marrow, Atypical Immunophenotype.

## Abstract

Background: Hairy Cell Leukemia (HCL) is an uncommon, indolent lymphoproliferative disorder of mature B lymphoid cells, accounting for 2% of all lymphoid tumors. The present study evaluated the clinical-hematological profile of HCL patients diagnosed at a single tertiary care center over a 11-year period. Methods: The retrospective observational study was done between October 2010 and September 2021. The relevant clinical and laboratory information were retrieved from hospital medical records and electronic databases. The statistical analysis was performed using version 23.0 of SPSS. Results: 66 (5.9%) of 1125 cases of chronic lymphoproliferative disorder were HCL. Splenomegaly was found in 47 (71.2%), hepatomegaly in 26 (39.5%), and lymphadenopathy in 17 (25.7%) of the cases. The mean hemoglobin, total leukocytes count, and platelets count were 8.04 g/dl, 6.76 X 109/L, and 77 X 109/L, respectively. Pancytopenia was detected in 40 cases (60.61 %). Bone marrow biopsies were majorly hypercellular and showed predominantly diffuse infiltration by atypical lymphoid cells. In two patients, initially thought of having refractory/hypoplastic anemia, the bone marrow biopsy and flow cytometry revealed HCL involvement.  42 cases of HCL underwent flow cytometry. CD20, CD 11c, CD 25 and CD 103 were positive in all the cases. The aberrant expression of CD5, CD10, and CD23 was found in frequencies of 5.71 %, 31.42 %, and 19.35%, respectively. In 40 cases for which follow-up information was available, there was full remission in 26 patients (65%), and later three showed relapse (7.5%) of which one died, and persistent leukemic activity in five (10%).  Eight patients (20%) died even before the initiation of treatment. One patient died within one month of therapy. No patient was examined for BRAF V600E mutation analysis. Conclusion: CD 10+ HCL was the most prevalent atypical immunophenotypic subgroup. Bone marrow biopsy and flow cytometry are crucial diagnostic tools to rule out hairy cell leukemia. However, BRAF V600E mutation analysis should be performed in cases with unusual presentation or resistance to treatment.

## INTRODUCTION

Hairy Cell Leukaemia (HCL) is an indolent lymphoproliferative disorder of mature B lymphoid cells with distinct morphological and immunophenotypic features^1^.

It is an uncommon entity that accounts for 2% of lymphoid tumours^1^, the term leukemic reticuloendotheliosis was used to designate it for the first time by Bouroncle et al. in 1958^2^.

HCL is now part of the subcategory of splenic B-cell lymphomas/leukaemias in the new World Health Organization Haematolymphoid Tumors 5 th edition (WHO-HAEM5), along with splenic marginal zone lymphoma/leukaemia (SMZL), splenic diffuse red pulp small B-cell lymphoma/leukaemia (SDRPL), and splenic B-cell with prominent nucleoli (SBLPN)^3^.

The neoplastic cells in HCL are typically small to medium in size, with oval nuclei and copious cytoplasm with distinctive circumferential hairy projections^4^. The hairy cell's immunophenotype resembles an active memory B cell and is characterized by bright coexpression of CD20 and CD22 as well as CD103, CD11b, CD25, and CD123^5^.

In over 95% of patients, the tumour cells have the somatic mutation BRAF V600E.

HCL is an indolent but progressive disease. It does not respond creditably to conventional lymphoma chemotherapy. Instead, they are sensitive to purine analogues such as cladribine and pentostatin and achieve complete remission in a significant portion of patients^6^.

Current study evaluates the clinical, haematological, and immunophenotypic features of HCL patients that have been diagnosed in our institution for over a decade, and briefly reviews the existing literature to share our experience.

## MATERIALS AND METHODS

The present retrospective observational study was carried out over an 11-year period, from October 2010 to September 2021. The cases were identified from the pathology database by looking for "Hairy cell leukaemia" in flow cytometry, bone marrow aspirate and biopsy reports. A total of 69 cases were identified, of which three were found to be hairy cell variants now called SBPLN.

Clinical details, radiological findings and laboratory results of the 66 classic HCL cases were compiled from hospital medical records and the electronic database. For morphological features and infiltration pattern analysis, Wright-stained peripheral blood, bone marrow aspirate smear, and Haematoxylin and Eosin-stained bone marrow biopsy slides were used. Reticulin stain was also put in some bone marrow biopsies, especially where increased bone marrow fibrosis was suspected. The eight-color flow cytometer FACS CANTO II was used for immunophenotyping. Bone marrow aspirate or peripheral blood collected in the ethylene diamine tetra acetic acid (EDTA) vacutainer was used as the sample. Cell preparation was done using the stain lyse wash method. The monoclonal antibody panel included CD45, CD19, CD20, CD5, CD10, CD11c, CD19, CD20, CD23, CD25, CD43, CD79b, CD103, CD200, FMC 7, surface immunoglobulin (Ig) light chains, and surface IgM. The cells were gated using CD19 PECy7 versus SSC.

The statistical analysis was conducted using SPSS version 23.0. The median and standard deviation (SD) were used to express quantitative variables, categorical variables were expressed as frequency. The Chi-square was utilized to compare categorical data, whereas the t-test was utilized to compare means. The p-values less than 0.05 were considered statistically significant.

## RESULTS

### Demography

66 (5.8%) of the 1125 cases of chronic lymphoproliferative diseases were found to be HCL. Weakness, stomach bloating, weight loss, fever, and bleeding were the primary symptoms. Splenomegaly was detected in 47 (71.2%) patients, with massive splenomegaly in 13 (19.7%) cases. In 26 (39.5%) of cases, hepatomegaly was seen. 17 cases (25.7%) of lymphadenopathy were found, with intrabdominal, cervical, axillary, and inguinal lymph nodes being the most frequent sites. Two of the four patients who underwent the Direct's Coomb's test had positive results.

### Laboratory

Mean haemoglobin (Hb) was 8.04 g/dl, and 32 (48.48%) of patients had severe anaemia. 6.76X10^9^/L was the mean total leukocyte count (TLC). 47 (71.21%) cases had leucopenia, while 11(16.67%) had leukocytosis.

The mean platelet count was 77 X10^9^/L. There were 58 (87.88%) cases of thrombocytopenia, with 24 (36.36%) of those cases having severe thrombocytopenia. In 54 (81.82%) cases, monocytopenia (less than 200/μl) was observed. Pancytopenia was present in 40 (60.61%), bicytopenia in 24 (36.36%) and isolated anaemia in two (3.03%) cases. Increased serum lactate dehydrogenase (sLDH) (>280 IU /L) was noted in 12 (18.18%) cases.

**Figure 1:**
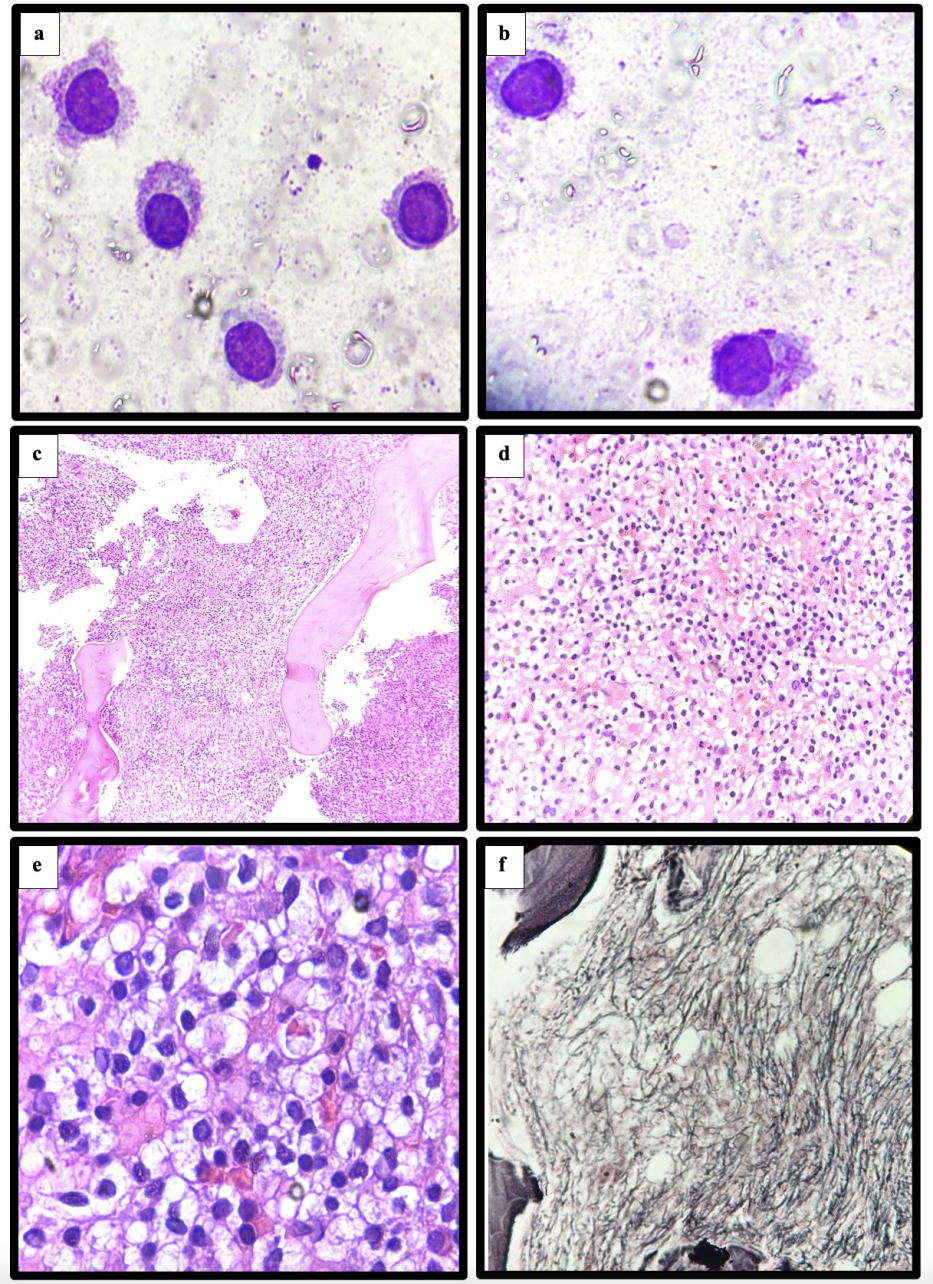
Morphological findings of hairy cell leukemia Peripheral (a) and Hemo-diluted Bone Marrow aspirate smears (b) showed many Hairy cells with oval to indented nuclei, homogenous chromatin, and abundant cytoplasm with circumferential cytoplasmic projections. Bone marrow biopsy shows diffuse infiltration by atypical lymphoid cells (c), with the fried egg appearance (d). High magnification shows widely spaced lymphoid cells with abundant cytoplasm and distinct cell borders. (e) Reticulin stain shows Diffuse and dense increases in reticulin fibers. (f) [Wright’s Giemsa stain, 1000 X (a & b); H&E, 40 X (c), 100X (d) & 1000 X (e) & reticulin stain, 400X (f)].

The hairy cells were in the peripheral smear or bone marrow aspirate of 51 (77.27%) cases. The atypical lymphoid cells were predominantly small to medium-sized and displayed round to oval to indented nuclei, coarse to homogenous ground glass chromatin, inconspicuous nucleoli, and abundant cytoplasm with circumferential hairy projections (Figure 1 a, b). Bone marrow biopsies were primarily hypercellular (40 cases, 60.61%) (Figure 1 c). The remaining 23 (36.36%) biopsies were normocellular, and three (3.03%) were hypocellular. The majority of bone marrow biopsies showed a diffuse pattern of atypical lymphoid cell infiltration (44 cases; 66.67%) (Figure 1 c-e), followed by interstitial (19 cases; 28.79%) and nodular (3 instances; 4.55%) patterns. Clinical and bone marrow aspirate results in two cases revealed refractory anemia/hypoplastic anemia. However, the bone marrow biopsies raised the suspicion of hairy cell leukemia infiltration and were later verified through flow cytometry. Reticulin stain varies from WHO grade 0 to grade 3 (Figure 1 e). The clinico-haematological and immune-phenotypic profile of HCL cases are described in Table 1.

**Table 1:** Clinical, Laboratory, Morphological and Flowcytometry Findings In Hairy Cell Leukemia Cases

Clinical & Laboratory Findings		Pattern of bone marrow infiltration	
Age (years)	53.1(range 29-70)	Diffuse	44/66(66.66%)
M: F ratio	15.5: 1	Interstitial	19/66 (28.79%)
Splenomegaly	47 /66 (71.2%)	Nodular	03/66 (4.55%)
Massive splenomegaly	13/ 66 (19.7%)	Reticulin grading	
Lymphadenopathy	17/66 (27.7%)	Grade 0, Grade 1, Grade 2, Grade 3	2/13, 1/13, 06/13, 04/13
Positive Direct Coomb’s test	2/4 ( 50 %)		
Mean Hemoglobin (in gm/dl)	8.04	Flowcytometry	
Total leucocyte count(X109/dl)	6.76	Marker	Frequency & Percentage (%)
Mean Platelet count (X109/dl )	77	CD 25	42/42 (100%)
Severe anemia (< 8 gm/dl)	32/66 (48.48%)	CD 11 c	42/42 (100%)
Leukocytosis (>11X109/dl)	11/66 (16.67%)	CD 103	42/42 (100%)
Leukopenia ( < 4 X109/dl)	47/66 (71.21%)	CD 123	6/7 (85.7%)
TLC in normal range	8/66 (12.12%)	CD 79 b	23/29 (79.3%)
Severe anemia (< 8 gm/dl)	32/66 (48.48%)	CD 45	26/26 (100%)
Thrombocyopenia	58/66 (87.88%)	CD 200	18/20 (90%)
Severe Thrombocytopenia (<50 X109/dl)	24/66 (36.36%)	CD 5	1/42 (2.39%)
Increased LDH (> 280 IU/ L)	12/66 (18.18%)	CD 23	6/42 (14.28%)
Pancytopenia, Bicytopenia, Isolated anemia	40/66 (60.61%), 24/66 (36.36%), 02/66 (3.03%)	CD 10	11/42 (26.19%)
		CD 43	11/15 (73.3%)
Peripheral Blood smear and Bone marrow examination findings		FMC 7	20/21 (95.2%)
Hairy cell in peripheral smear	51/66 (77.27%)	S IgM	12/14 (85.7%)
			
Bone marrow cellularity		Light Chain Restriction	
Hypercellular, Hypocellullar, Normocellular	40/66 (60.61%), 03/66 (3.03%), 23/66 (36.36%)	Kappa, Lambda, Equivocal, Negative	10/32 (31.25%), 15/32 (46.87%), 2/32 (6.25%), 5/32 (15.62%)

### Immunophenotyping

**Figure 2:**
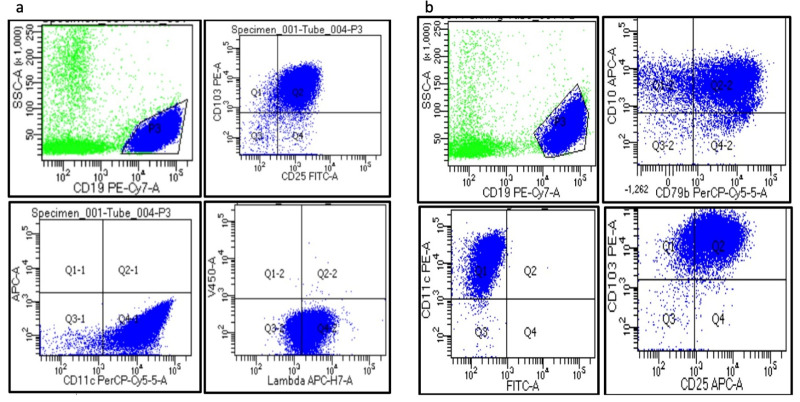
Flowcytometry showed coexpression of CD11c, CD25, CD103 with lambda light chain restriction in the neoplastic cells; (a) and in other case aberrant expression of CD 10 was noted (b)

Flow cytometry was done in 42 cases. Classical HCL immunophenotypic markers were expressed in all the cases with positivity for CD 20, CD22, CD11c, CD25 and CD103. CD 123, CD 200, FMC7 and CD 79b were positive in 85.7%, 90.0%, 95.2%, and 79.3% of cases, respectively (Figure 2 a). Aberrant expression of CD5, CD10 (Figure 2 b) and, CD23 was encountered in 2.39%, 26.19 % and 14.28 %, respectively (Table 1). There was no discernible difference in the clinicopathological profile between the two frequently occurring atypical immunophenotypic groups, i.e., CD 10 positive HCL (CD 10+ HCL) and CD 23 positive HCL (CD 23 +HCL), and the typical immunophenotypic group (Table 2). Of thirty-two cases evaluated for light chain restriction, ten (31.25%) exhibited kappa chain restriction, 15 (46.87%) cases showed lambda chain restriction and equivocal in two cases (6.25%) and negative in five (15.62%). Surface IgM was weak to moderate positive in 12 of 14 cases (85.71%). The diagnosis was made based on morphology and with the use of IHC in the fourteen cases where the bone marrow aspirate was diluted and flowcytometry could not be performed. Based on CD 20, PAX5, and CD 79b, a possible diagnosis of hairy cell leukaemia was made and annexin A1 IHC was recommended because it was not available in our hospital's setup. Six patients had annexin A1 tests performed outside and turned out to be positive supporting the HCL diagnosis. Ten cases had no available IHC or flow cytometry data since they were referral cases, diagnosed elsewhere, and only had their bone marrow aspirates and biopsies examined at our hospital.

**Table 2:** Comparison of Classical HCL versus CD10+ HCL and Classical HCL versus CD 23+cases

	Classical (n=24)	CD 10 (n=11)	P value	Classical (n=24)	CD 23 (n=6)	P value
Age (mean)	54.6 ±8.1	47.91+10.11	0.53	54.6 ±8.1	50.83+14.414	0.413
Hemoglobin (mean) (Hb in gm/dl)	7.75±2.36	8.63+3.38	0.766	7.75±2.36	6.23+2.16	0.165
Severe anemia	11 (45.8%)	4 (36.4%)	0.599	11 (45.8%)	5 (83.3%)	0.100
TLC (mean) (X109/dl)	9.74±18.42	5.55+6.75	0.472	9.74±18.42	16.28+25.25	0.476
ANC (mean) (/ul)	1543	1047	0.457	1543	853.17+660.94	0.437
Neutropenia	14 (58.3%)	9 (81.8%)	0.174	14 (58.3%)	5 (83.3%)	0.256
Platelet (mean) (X109/dl)	71.33±55.44	60.45+45.20	0.573	71.33±55.44	71.00+66.97	0.990
Severe thrombocytopenia (<50000/ul)	11 (45.8%)	4 (36.4%)	0.599	11 (45.8%)	3 (50%)	0.855
Absolute hairy cell count (/ul)	5757	3210	0.617	5757	12201+21943	0.425
Hairy cell count (>5000/ul)	6 (25.0%)	2 (18.2%)	0.656	6 (25.0%)	3 (50%)	0.232
Cytopenia: Pancytopenia, Bicytopenia, Isolated anemia	15 (62.5%), 9 (37.75%),0 (0%)	5 (45.5%), 6 (54.5%), 0 (0%)	0.344	15 (62.5%), 9 (37.75%),0 (0%)	2 (33.33%), 3 (50%), 1 (5.6%)	0.086
Monocytes (mean) (/ul)	383.04±740.43	421.55+659.95	0.652	383.04±740.43	436.17+471.75	0.869
Monocytopenia (<200/ul)	17 (70.8%)	5 (45.5%)	0.149	17 (70.8%)	2 (33.33%)	0.088
LDH (median) (IU/L)	231.53±115.96	232.28±66.86	0.986	231.53±115.96	202±255	0.687
Increased LDH (>280 IU/L)	4 (16.7%)	3 (27.3%)	0.466	4 (16.7%)	2 (33.33%)	0.361
Splenomegaly	20 (83.3%)	10 (90.9%)	0.552	20 (83.3%)	5 (83.5%)	1.000
Massive splenomegaly	13 (54.2%)	4 (36.4%)	0.328	13 (54.2%)	2 (33.33%)	0.361
Hepatomegaly	10 (41.7%)	45.5%)	0.833	10 (41.7%)	1 (16.7%)	0.256
Lymphadenopathy	11 (45.8%)	2 (18.2%)	0.124	11 (45.8%)	1 (16.7%)	0.192
Bone marrow cellularity: Hypercellular, Normocellular, Hypocellular	12/24 (50%), 12/24 (50%), 0 (0%)	4/11 (36.4%), 6/11 (54.5%), 1/11 (9.1%)	0.284	12 (50%), 12 (50%), 0 (0%)	6/6 (100%), 0 (0%), 0 (0%)	0.025
Hairy cell percentage in BM (%)	54.83±24.31	65.18+19.43	0.224	54.83±24.31	51.00+22.44	0.729

### Treatment and follow up

**Figure 3:**
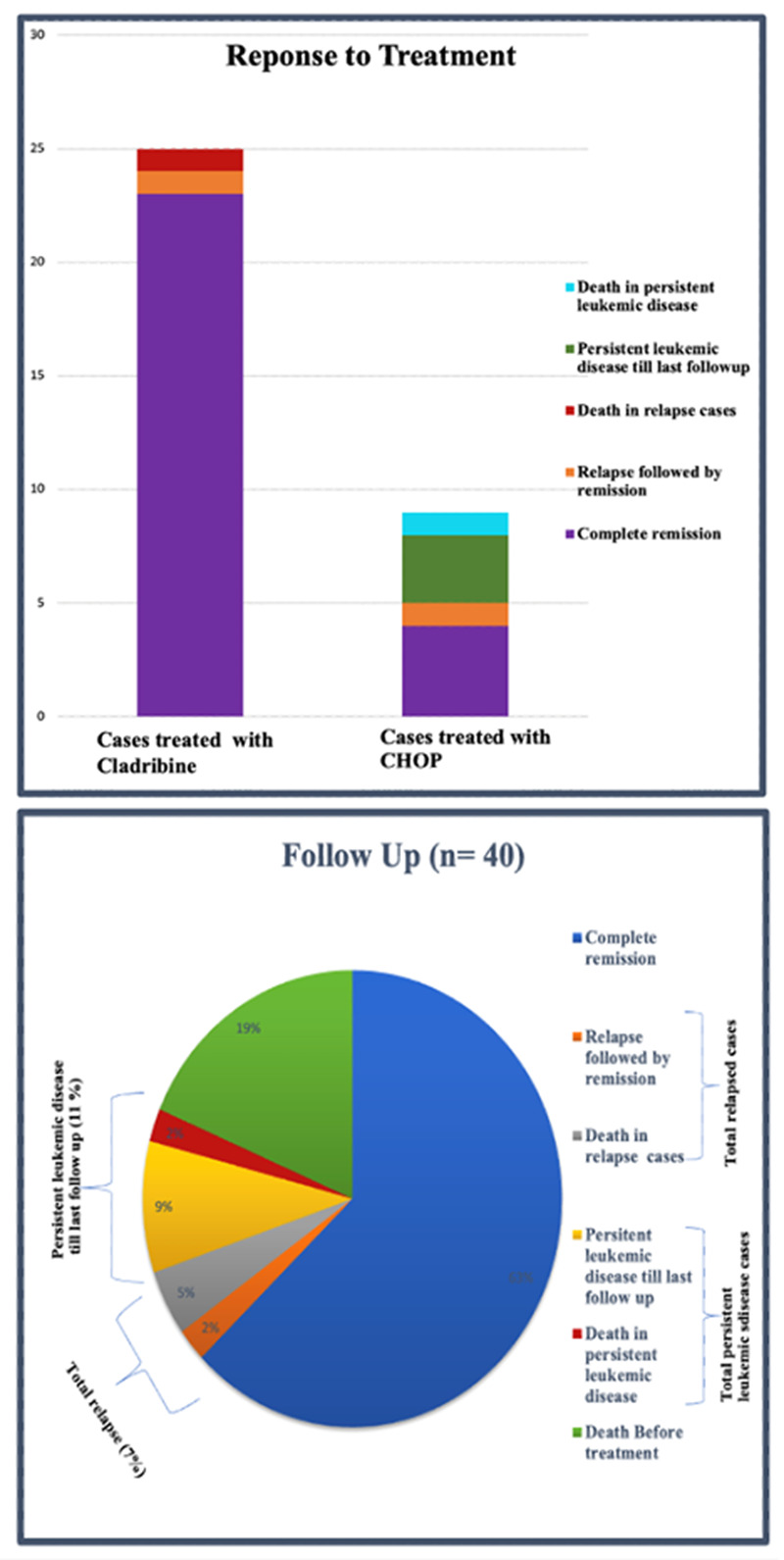
Stacked column and pie-chart showing the Therapy response and overall follow up

A total of 40 patients opted for treatment at our institution, with a mean follow-up of 33.13 months (ranging from two months to 109 months). Five patients died even before the initiation of treatment due to neutropenia and acquired infections. Three died at the time of the covid pandemic in 2020 due to acute respiratory syndrome. Twenty-two patients received cladribine as the first line of treatment. All patients achieved complete remission after one cycle. Two cases relapsed after one and three years of remission, of which the former patient died due to disease-related complications, and the latter achieved remission on the second dose of cladribine and was alive until 10 years of follow-up since the time of diagnosis. Neutropenia was taken care of in all the patients by administration of recombinant growth factors. Ten patients, due to lack of affordability, opted for cyclophosphamide, hydroxydaunorubicin, oncovin (vincristine), prednisolone (CHOP) therapy. One of them died within a month of therapy. Four patients achieved complete remission, of which one relapsed after one year. Five never achieved remission until their last follow-up (Figure 3). BRAF V600E mutation analysis was not done in any patient due to our institution's unavailability of the test.

## DISCUSSION

In contrast to the 2% reported in the literature, the HCL makes up a greater proportion of lymphoid leukemia in our study^7,8^. However, the higher prevalence has been described in Indian literature, in part because of referral bias^4,9-11^.

The age range was 29-70 years, and the mean was 53.19 years. Male: Female ratio was 15.5:1, which concorded with the previous studies^10^. The youngest patient was 29 years old. Gupta et al. have described 22 years old as the youngest in the Indian subcontinent^5^.71.2% of cases presented with splenomegaly, almost similar to Somasundaram et al., 2015^4^. The incidence of HCL without splenomegaly in various series has ranged from 0 to 30%; in our study, it was 28.8%. The highest rate (40%) was described by Venkatesan, 2014 et al^12^.

Lymphadenopathy was reported in studies ranging from 8.5% to 30%, and it made up 25.7% of the cases in our study^5^. Refractory anaemia or hypoplastic anaemia was suspected in two cases. Both of them had pancytopenia when they were first diagnosed, but neither had splenomegaly or lymphadenopathy. The diagnosis of hypoplastic anaemia was confirmed by relative lymphocytosis in bone marrow aspirate smears. However, a bone marrow biopsy revealed hypocellular marrow with a fried egg appearance that showed lymphoid cell infiltration. A diagnosis of hairy cell leukaemia was suspected and later confirmed on flow cytometry. Likewise, Gupta R et al. described the atypical presentation of HCL, clinically and morphologically mimicking aplastic anaemia^5^.Using flow cytometry, a hairy cell leukaemia diagnosis was later confirmed. The unusual presentation of HCL, which morphologically and clinically mimics aplastic anaemia, was also documented by Gupta R et al^5^.

Peripheral pancytopenia and monocytopenia are two typical HCL manifestations. However, leucocytosis was detected in 16.67% of patients, and 18.18% of cases lacked monocytopenia, as has previously been noted in other publications^4,5,9-11^.

The classical HCL immunophenotypic markers were expressed in all the cases. Aberrant immunophenotypic expression of CD5 (2.39%), CD10 (26.19%) and CD 23 (14.28%) were noted in 18 of 42 cases (42.86 %). The cases of atypical immunophenotype were a little higher than that of Chen et al (34%). Contrary to Chen et al and Gupta et al. where CD 23 + immunophenotype was the major atypical immunophenotype, in our case, CD 10 was the most prevalent atypical immunophenotype in our study, comprising 26.19% of all flowcytometry-analyzed cases^5,6^. The atypical immunophenotypic subgroups did not exhibit any substantial clinicopathological changes, similar to other reported cases^9^.Similar to other reported cases, we also didn’t get any significant clinicopathological variations among the atypical immunophenotypic subgroups^6^. The atypical immunophenotypic group has no clinicopathological significance, and more studies are required to see their prognostic and diagnostic significance.

Numerous morphological imitators of hairy cell leukaemia exist, including splenic B cell lymphoma/leukaemia with prominent nucleoli (HCL variant), splenic diffuse red pulp small B lymphoma, splenic marginal zone lymphoma, chronic lymphocytic leukaemia and mantle cell lymphoma. The use of flow cytometry aids in establishing a definite diagnosis. In hairy cell leukaemia, all four CD11c, CD25, CD103, and CD123 are frequently positive. Additionally, unlike other differentials, atypical lymphoid cells in HCL show positive immunohistochemistry for Annexin A1. CD 25 and CD 123 are frequently negative in SBLPN. SDRPL are frequently positive for CD 20 and IgG and negative for CD 25, CD 103, CD 123, and CD 11c. SMZL cases show variable positivity for CD 25, CD 11c and CD 123, and are typically negative for CD103 and annexin A1. SMZL tumour cells express surface IgM and IgD in contrast to HCL, where surface IgG is typically seen^13^.

There is no widely agreed upon staging system for HCL. Lymphadenopathy is uncommon (<9%) and the incidence is common at relapse and associated with inferior responses and outcomes^14^. Treatment of HCL patients has advanced over the years with the use of interferon in 1980, to use of purine analogues in the 1990s, a monoclonal antibody in the 2000s, and BRAF inhibitors in the current decade. The main aim of initial treatment is to achieve complete remission; defined as recovery of peripheral blood counts, Hb>11gm/dl, ANC>1500, platelet count >100000/ul, regression of splenomegaly, morphological absence of disease in the bone marrow and peripheral blood. Purine nucleoside analogues (cladribine, pentostatin) with or without rituximab consolidation remain the standard front-line treatment for HCL with durable response seen in >90% of patients with PFS ranging from 9-11 years. Among purine analogues, Cladribine is the preferred agent because of its ease of administration with a defined schedule. Two schedules for cladribine administration are available: Cladribine= 0.1mg/kg for 7 days as a continuous infusion^15,16^ and Cladribine = 0.14 mg/kg IV OD over 5 days^17^. Many patients treated with purine analogues will achieve complete remission, with up to 20% having a partial response and only 4% having the stable or progressive disease^15,18,19^. Many patients remain disease free for prolonged periods with only 14-20% relapsing at 30 months and 36% at 9.7 years^20^. OS varies between 79-87 % at 12 years in different series. For Patients of HCL resistant to purine analogues, it is recommended to switch to alternative purine analogues or can be offered BRAF inhibitors with rituximab.

Virtually all cases of HCL exhibit somatic BRAF V600E mutation leading to constitutive activation of the RAF-MEK-ERK signaling pathway and enhanced survival. This forms the basis of the use of BRAF inhibitors in patients with HCL. Off-label use of vemurafenib in combination with rituximab is reserved for those patients who have primary refractory disease or those who relapse within 24 months. If used alone, BRAF inhibitors induce only partial response transiently. Addition of rituximab results in faster and deeper responses with longer remissions as shown in one phase 2 trial^21-23^.

**CONCLUSION**

Given the rarity of hairy cell leukaemia and the potential for misleading presentations like refractory anaemia and the possibility that typical hairy cells may not be visible in peripheral blood smears, a bone marrow examination, particularly bone marrow biopsy, is a crucial diagnostic procedure to rule out involvement. CD 10 + HCL was the most common atypical immunophenotypes. Aberrant immune phenotypes hold no clinicopathological significance. In most cases, the typical morphology and distinctive flowcytometry or immunohistochemistry findings are enough to make the diagnosis, but in cases where the patient is resistant to treatment or presents in an unusual way, BRAF V600E mutation analysis should be performed to make the diagnosis or to begin treatment with anti-BRAF V600E mutation.
